# Reviewer acknowledgement 2013

**DOI:** 10.1186/1710-1492-10-5

**Published:** 2014-02-03

**Authors:** Richard Warrington

**Affiliations:** 1 University of Manitoba, Manitoba, Canada

## Abstract

**Contributing reviewers:**

The editors of *Allergy Asthma & Clinical Immunology* would like to thank all of our reviewers who have contributed to the journal in Volume 9 (2013).

## 

Nils Aberg

Sweden

Ritesh Agarwal

India

Reza Alizadehfar

Canada

Samuel Arbes

United States of America

Nilgun Atakan

Turkey

Meghan Azad

Canada

Claus Bachert

Belgium

Sami Bahna

United States of America

Anand Balasubramani

United States of America

Brian Blakley

Canada

Carlos Blanco

Spain

Robert Boyle

United Kingdom

Knut Brockow

Germany

Jean-Marie Bruzzese

United States of America

Anette Bygum

Denmark

Scott Cameron

Canada

Stuart Carr

Canada

Jamila Chakir

Canada

Edmond Chan

Canada

Salvatore Chirumbolo

Italy

Don Cockcroft

Canada

Mitch Cohen

United States of America

Linda Cox

United States of America

Maria Dalamaga

Greece

John Dean

Canada

Deepak Deshpande

United States of America

Tony Dubois

Netherlands

Audrey Dunn Galvin

Ireland

Bernadette Eberlein

Germany

Aarif Eifan

United Kingdom

Rashika El Ridi

Egypt

Anne Ellis

Canada

Adegoke Falade

Nigeria

Daniela Fenoglio

Italy

Charles Frankish

Canada

Abdelilah Gounni

Canada

Paolo Gresele

Italy

Eyal Grunebaum

Canada

Michelle Halbrich

Canada

Kent HayGlass

Canada

Jonathan Hourihane

Ireland

Nedim Ince

United States of America

Komei Ito

Japan

Sushil Kabra

India

Rhoda Kagan

Canada

Sandeep Kapur

Canada

Fotini Kavadas

Canada

Paul Kevin Keith

Canada

Atsushi Kitani

United States of America

Ester Klaassen

Netherlands

Rengin Kosif

Turkey

Mitja Kosnik

Slovenia

Astrid Kruizinga

Netherlands

Rajesh Kumar

United States of America

Dinakantha Kumararatne

United Kingdom

Ying Lai

United States of America

Tracy Langkilde

United States of America

Søren Thor Larsen

Denmark

Ian Lewkowich

United States of America

Per Lidman

Canada

Chi-Chen Lin

Taiwan

Allan Linneberg

Denmark

Je-Ruei Liu

Taiwan

Scott Lorch

United States of America

Bruce Mazer

Canada

Lawrence Mbuagbaw

Cameroon

Christine McCusker

Canada

Chris Mody

Canada

Dumitru Moldovan

Romania

David William Moote

Canada

Thomas Murphy

United States of America

Kari Nadeau

United States of America

Bing Ni

China

Jean-Francois Nicolas

France

Akio Niimi

Japan

Haruhiko Ogawa

Japan

Mitsuhiro Okano

Japan

Kimihiro Okubo

Japan

Cevdet Özdemir

Turkey

Evy Paulsen

Denmark

Cesar Picado

Spain

Thomas Platts- Mills

United States of America

Paul Charles Potter

South Africa

Gregory Rex

Canada

Rachel Robison

United States of America

Monica Ruiz-Garcia

United Kingdom

Franca Rusconi

Italy

Joseph Sakshaug

Germany

Bob Schellenberg

Canada

Peter Schmid-Grendelmeier

Switzerland

Carrock Sewell

United Kingdom

Ashok Shah

India

Christopher Shaw

Canada

William Shearer

United States of America

Michael Shields

United Kingdom

Fanny Silviu-Dan

Canada

Donald Stark

Canada

Hariharan Subramanian

United States of America

Masaya Takemura

Japan

Susan Tarlo

Canada

Luis Teran

Mexico

Simon Francis Thomsen

Denmark

Euan Tovey

Australia

Peter Vadas

Canada

Steven Valles

United States of America

Johannes C van der Wouden

Netherlands

Timothy Vander Leek

Canada

Susan Waserman

Canada

Wade Watson

Canada

Anges Yadouleton

Benin

